# Economic analyses to support decisions about HPV vaccination in low- and middle-income countries: a consensus report and guide for analysts

**DOI:** 10.1186/1741-7015-11-23

**Published:** 2013-01-30

**Authors:** Mark Jit, Carol Levin, Marc Brisson, Ann Levin, Stephen Resch, Johannes Berkhof, Jane Kim, Raymond Hutubessy

**Affiliations:** 1 Modelling and Economics Unit, Health Protection Agency, 61 Colindale Avenue, London NW9 5EQ, UK; 2 Department of Infectious Disease Epidemiology, London School of Hygiene and Tropical Medicine, Keppel Street, London WC1E 7HT, UK; 3 PATH, 2201 Westlake Avenue, Suite 200 Seattle, WA 98121 USA; 4 Centre de recherche du CHU de Québec, Hôpital Saint-Sacrement, 1050 Chemin Sainte-Foy, Québec, G1S 4L8, Canada; 5 Department of Infectious Disease Epidemiology, Imperial College, London, UK; 6 Independent Consultant, 6414 Hollins Dr., Bethesda, MD 20817 USA; 7 Center for Health Decision Science, Harvard School of Public Health, 718 Huntington Ave, Boston, MA 02130, USA; 8 Department of Epidemiology and Biostatistics, VU University Medical Centre, PO box 7057, 1007 MB Amsterdam, The Netherlands; 9 Initiative for Vaccine Research, World Health Organization, 20 Avenue Appia, CH-1211 Geneva, Switzerland

**Keywords:** Human papillomavirus, vaccination, low- and middle-income countries, economic evaluation.

## Abstract

Low- and middle-income countries need to consider economic issues such as cost-effectiveness, affordability and sustainability before introducing a program for human papillomavirus (HPV) vaccination. However, many such countries lack the technical capacity and data to conduct their own analyses. Analysts informing policy decisions should address the following questions: 1) Is an economic analysis needed? 2) Should analyses address costs, epidemiological outcomes, or both? 3) If costs are considered, what sort of analysis is needed? 4) If outcomes are considered, what sort of model should be used? 5) How complex should the analysis be? 6) How should uncertainty be captured? 7) How should model results be communicated? Selecting the appropriate analysis is essential to ensure that all the important features of the decision problem are correctly represented, but that the analyses are not more complex than necessary. This report describes the consensus of an expert group convened by the World Health Organization, prioritizing key issues to be addressed when considering economic analyses to support HPV vaccine introduction in these countries.

## Background

The World Health Organization (WHO) recommends that cost-effectiveness be considered before human papillomavirus (HPV) vaccination is introduced in national programs [1]. However, many low- and middle-income countries (LMICs) lack the technical capacity and accurate empirical data to develop and parameterize *de novo *models of complex interventions such as HPV vaccination [2-5]. Given these constraints, LMIC decision-makers may want to apply or adapt a previous economic evaluation conducted in a different country. The practice of adapting existing models is common: one systematic review of economic evaluations of HPV vaccination found that 35 of 58 relevant articles were adaptations of previous models [6]. However, most existing analyses (44/58) were set exclusively in high-income countries (HICs). Hence, analysts in LMICs who want to understand whether they should adapt a previous model, develop a new one, or not conduct an economic evaluation at all have fewer examples and guidance on which to rely.

Furthermore, analysts often face a dilemma in choosing which models to develop or adapt. On the one hand, simpler models are easier to parameterize, adapt, and interpret; however, such models are designed to answer a limited range of questions, and can be misleading if used to address more complex issues [7,8]. On the other hand, models that are equipped to address more complex issues may require expertise or data that may not be available in that country.

In addition to cost-effectiveness results, decision-makers may need to know the financing requirements and affordability of vaccine introduction to support financial planning and forecasting. Such considerations are informed by models that comprehensively capture the programmatic costs and logistic considerations involved in national scale-up of vaccine introduction [9], but are less focused on reproducing the natural history and epidemiology of HPV-related diseases.

A further challenge is translating economic results into conclusions that are useful to decision-makers. In particular, decision-makers need to understand the type of policy questions that economic models can address, and the appropriate caveats around model conclusions (such as data shortcomings and model uncertainty).

To address these questions, the WHO convened a panel of modelers and economists to develop guidance for analysts based in LMICs who advise policy-makers on HPV vaccination (but based on principles applicable to all countries). Panel members held several meetings to outline issues, then agreed on a summary. Each member then drafted sections of the guidelines, then received comments from all other participants. Comments were collated, harmonized and further reviewed by all participants until complete consensus was reached. Full details of guideline development are available in the Appendix.

## Question 1: Is an economic analysis needed?

Three ways to use economic analyses to inform decisions around HPV vaccination are: 1) conducting no structured analysis, 2) borrowing insights from analyses in other settings, and 3) conducting a *de novo *country-specific analysis using an economic model.

The option of not conducting any analysis is particularly attractive in LMICs with limited analytical capacity. However, such countries may also have the greatest need for evidence, because poor allocation of funding at the margin has greater health consequences when funds are limited.

Policy-makers may also consider using existing analyses and insights from other settings. When analytical results are stable across settings, conducting new setting-specific studies may add little value. For example, model conclusions about vaccinating girls before onset of sexual activity have been similar across HICs [7,8]. There have been fewer results from LMICs, but existing analyses suggest that HPV vaccines need to be priced much more competitively in LMICs than in HICs for vaccination to be cost-effective [6,10].

Vaccine price, HPV prevalence, and uptake of cancer screening and treatment are key drivers of the cost-effectiveness of HPV vaccination in LMICs [6,10]. Hence, studies investigating similar policy questions in settings where such parameters are similar can be adapted to a new setting. Many parameters that vary across settings (such as unit costs) are usually easy to adjust for in economic models. Hence, before deciding whether to conduct a *de novo *analysis, decision-makers should consider existing analyses, investigate the main drivers of results in similar countries, and appraise the extent to which these analyses are suited for their own country. Ideally, such a process should be transparent and unbiased, using best-practice methodology for evidence review and synthesis [11].

## Question 2: Should analyses address costs, epidemiological outcomes or both?

Once a decision is made to conduct an economic evaluation, the type of analysis needs to be chosen. First, a clear, answerable policy question is defined [12]. This will determine 1) which costs and/or health outcomes (for example, cervical-cancer incidence or disability-adjusted life years (DALYs) lost) are considered, 2) what the intervention and comparator under evaluation are, 3) who the target audience is, and 4) what methodological options (such as time horizon and economic perspective) are chosen.

Analyses involving costs alone usually use primary data from demonstration projects and other studies exploring different HPV vaccine delivery programs [9]. These are useful for several reasons. Firstly, vaccine purchase costs are often borne by external donors who make decisions based on their own analyses. However, national decision-makers still need to investigate whether vaccine delivery is affordable in the short term and sustainable in the long term. Secondly, if vaccination is introduced, planners need to allocate resources for the financial costs of introducing the vaccine. Thirdly, these data inform cost minimization studies that address questions such as optimal delivery strategy (school-based, health facility-based, using national immunization days, or a combination). They also inform budget impact analyses, and are essential inputs into full economic evaluations such as cost-effectiveness analyses.

Alternatively, models may project the effects of vaccination on disease burden [13], using measures such as cervical-cancer morbidity, mortality, or DALYs. These studies are essential inputs for cost-effectiveness analyses, but are also useful in their own right to inform surveillance and to understand emerging post-vaccination trends such as changes in the average age of infection, waning vaccine effectiveness, herd immunity, and potential type replacement. They can also show if vaccination is equitable in terms of how its effects are distributed by income strata, geographical location, ethnic group, and gender.

For setting priorities, full economic analyses considering both costs and outcomes are needed. Such analyses address whether HPV vaccination is worth the investment compared with either current practice such as existing cervical-cancer screening and treatment [14] or doing nothing [15]. They can also address more detailed questions such as the choice of vaccine (bivalent or quadrivalent), target ages for routine and catch-up vaccination, inclusion of boys in the HPV vaccination program [16], and revising screening policies to optimize synergies with vaccination [8].

In HICs, economic models often inform evidence-based priority-setting discussions through National Immunization Technical Advisory Groups (NITAGs) [17]. In LMICs, priority-setting discussions are still relevant, but may not be held because of lack of capacity to perform local evaluations and/or the absence of advisory groups to interpret results. In addition, decisions are often driven by donors such as international funding agencies or individual donor countries, so spending priorities are set by the international community. Therefore, more pragmatic analyses such as budget impact analyses focusing on cost savings, affordability, and sustainability may be more relevant in these countries [18].

## Question 3: If costs are considered, what sort of analysis is needed?

An economic analysis involving costs involves the following steps.

1) Plan a data-collection study, which can be either retrospective or prospective.

2) Choose between calculating financial or economic costs (or both).

• Financial costs are the value of resources to the payer (such as the Ministry of Health), and are used to assess affordability. They include actual resources purchased such as injection supplies, outreach and daily allowances, and funds for training and developing new communication materials.

• Economic costs comprise the opportunity costs of all outlays, including resources already paid for or owned by the Ministry of Health, and other sources of financing, such as the salaries of health personnel, vaccines paid for by partners, and donated equipment or services such as volunteer time. They give a more complete picture of resources that are tied up in the provision of the new vaccine and their opportunity costs, and should be used if a cost-effectiveness or cost-benefit analysis is to be conducted. They are also used to evaluate sources of required financing for introduction and scale-up, and to assess the feasibility of providing the necessary health system requirements on a sustainable basis. They also give a better assessment of the share of costs financed by different partners including external partners, because they include the value of donated goods.

3) Choose between calculating costs using an ingredients approach, or using average costs.

• An ingredients approach estimates the quantity and cost of each individual resource required by a program [19]. This is the preferred approach to evaluate the value of resources used to introduce HPV vaccination because the service-delivery strategies used are different from those used for infant vaccines.

• Average costs based on administrative returns or tariffs are often used in economic evaluations in HICs, on the basis that delivery costs are small compared to the cost of vaccine purchase, and hence inaccuracies in their estimation are less important. However, such assumptions may not hold in many LMICs.

4) Choose between incremental costs (the additional costs that occur with the HPV vaccination (or other intervention)) or all costs, including shared costs. For example, the transport costs may be shared with other vaccines, so would not be fully included in an incremental analysis. Incremental cost analyses are typically used for cost-effectiveness and cost-benefit analyses. However, for partial economic analyses, total cost analyses can inform comparisons between different strategies.

5) If vaccination is being compared with screening and treatment, estimate the value of resources used for screening, diagnosis, and treatment of neoplasias. Even in health systems without organized screening, there is a need to account for the cost of providing cancer treatment and palliative care.

6) Distinguish between recurrent or operational costs (those lasting less than a year) and capital costs (lasting longer than a year), so that the value of capital goods can be spread over a longer period [19].

7) Identify start-up costs needed in the first year of vaccination, such as introduction costs (microplanning, initial training, and initial social mobilization material development) and capital goods (additional cold chain equipment, vehicle requirements, and incinerators).

## Question 4: If outcomes are considered, what sort of model should be used?

Model choice should be driven by the policy questions to be answered. The simplest models (termed 'proportionate outcomes static models'; Table 1), do not capture indirect benefits of vaccination (herd immunity), but can still provide conservative estimates of the cost-effectiveness of vaccinating girls before they start sexual activity. They also do not capture changes in sexual activity, screening behavior, or demographics.

**Table 1:** Types of models involving health outcomes, from least to most complex.

Model type	Features	Data requirements		
		
		Demography	Epidemiology	Clinical
Static proportionate outcomes model	Reduction in cervical cancer due to vaccination is the product of effective vaccine coverage and cervical-cancer incidence caused by vaccine-type HPV	Population structure, birth and death rates	Cervical-cancer incidence, mortality and proportion caused by HPV 16/18, by age	None

Static progression model	Represents the natural history of disease from HPV infection to cervical cancer, usually in a single birth cohort	Population structure, birth and death rates	Cervical-cancer incidence, mortality and proportion caused by HPV 16/18, by age; female HPV prevalence and clearance by age	Uptake and efficacy of screening, diagnosis and treatment of cancer and pre-cancer

Transmission dynamic model	Captures HPV transmission from infected to susceptible individuals in the entire population	Population structure, birth and death rates, sexual behavior	Cervical-cancer incidence, mortality and proportion caused by HPV 16/18, by age; HPV prevalence and clearance by age and sex	Uptake and efficacy of screening, diagnosis and treatment of cancer and pre-cancer

Hybrid model	Uses a static progression model to capture disease natural history, and a dynamic model to capture infection transmission	Population structure, birth and death rates, sexual behavior	Cervical-cancer incidence, mortality and proportion caused by HPV 16/18, by age; HPV prevalence and clearance by age and sex	Uptake and efficacy of screening, diagnosis and treatment of cancer and pre-cancer

Proportionate outcomes models assume that cervical-cancer incidence does not change in the absence of vaccination, so should not be used in countries in which cervical screening has recently or will shortly be introduced. Ignoring the potential reduction in cervical-cancer incidence resulting from screening would bias the results in favor of HPV vaccination. Such situations require the use of 'static progression models' (Table 1), which model the natural history of disease from HPV infection to cervical cancer, and the effect of screening on disease progression.

Questions such as the incremental cost-effectiveness of vaccinating boys, the optimal age of routine and catch-up vaccination, and the optimal combination of screening and vaccination, should be examined using 'transmission dynamic models' (Table 1) which capture indirect effects of vaccination [8,20]. Such models are also required if evidence is needed on the magnitude and timing of the benefits of vaccination.

Static models overestimate the incremental benefit of vaccinating boys in addition to girls [20]. This is because they do not capture the herd protection males already receive from female-only HPV vaccination programs. Hence, a dynamic model is needed to show that male vaccination is cost-effective, although a static model may be used to confirm that vaccinating boys is not cost-effective at all, as the results under the dynamic model will be less favorable than under the static model. Furthermore, it is unclear whether using a static model will produce conservative estimates for catch-up strategies. This is because the potential for incremental gains in the catch-up cohorts may be smaller under a dynamic framework where some vaccinated individuals would have been indirectly protected through herd immunity [8].

Simple static cohort models are usually difficult to adapt into the dynamic models. Hence, some studies have captured herd immunity using hybrid models, which use dynamic models to represent transmission, and static models to represent disease progression and screening (Table 1) [15,16,21]. Hybrid models can address the same policy questions as dynamic models, but can be incorporated as add-on modules to static models. Thus, end users can begin with the simpler model, and then use the hybrid module for more complex questions. The disadvantage of this approach is that the way in which dynamic model predictions inform the static model can lack transparency.

Countries or donors may need to choose between the competing HPV vaccines. Few modeling studies have directly compared bivalent and quadrivalent vaccines, and none of these studies have been carried out in LMICs. Studies in HICs suggest that quadrivalent vaccination is more cost-effective than bivalent vaccination if they are equally priced, because of the additional benefits of preventing genital warts [22-24]. In many LMICs however, preventing cancer may be the main or sole priority for HPV vaccination, so bivalent vaccination may have an advantage because of its better cross-protection against non-vaccine oncogenic HPV types [25]. Such questions should be addressed using multi-type transmission dynamic models because 1) static models do not capture the picture of wart prevention in males as a result of vaccinating females, and 2) multi-type models are needed to accurately capture the different cross-protective effects of the two vaccines.

## Question 5: How complex should the analysis be?

Model types range from simple 'proportionate outcomes' spreadsheet models to complex dynamic transmission models (see Table 1). Complex models may generate more accurate results and address more questions, but require additional data, time, effort, and expertise. Using them may require dependence on external consultants or consume rare local expertise. However, they may also engage local experts with strong analytical skills in the public health policy-making process.

The first consideration must be to ensure that model results increase the probability of a decision aligned with the decision-maker's preferences. Oversimplified models may make poor predictions, leading to worse policy choices than would have occurred in the absence of model-based evidence. Hence, sometimes only a sophisticated model is appropriate. For example, if a static model (which ignores herd immunity) suggests that vaccination is not cost-effective, then vaccination should not be rejected without first testing the conclusion using a dynamic model that will capture more benefits. Similarly, investigating the relative value of bivalent and quadrivalent vaccines, or the precise upper age limit for catch-up vaccination, require sophisticated models calibrated with good data.

A second consideration is the trade-off between the incremental informational value and additional time/effort needed for a more sophisticated modeling approach. A model should be as parsimonious as possible; it should capture effects vital to understanding the policy questions (such as herd protection when examining the effects of vaccinating boys on the incidence of disease in girls), but equally should not have unnecessary detail [26]. Determining whether particular effects are important for decision-making may require a more complex model to investigate whether the results of the simpler model are biased. Often, analysts can draw on a repertoire of simple model structures that have been validated against more complex models and peer-reviewed. For instance, a range of HPV models were recently compared using a standardized input dataset [6]. Analysts can also draw on methodological work investigating the effect of different simplifications to model structure [20]. However, methodological investigations in middle- and high-income settings may not represent behavioral determinants and disease co-factors in low-income settings.

The third consideration is the data requirement. Even simple models require demographic, epidemiological, clinical, and economic data (Table 1). Ideally, these should be local data from large population-based cohorts or adequately powered trials. When unavailable, less reliable data sources must be used, such as data from cancer registries and health care utilization reports (which may be incomplete and hence biased), or data extrapolated from other countries. Complex models may have greater data requirements, so data shortcomings may compromise their benefits by introducing additional uncertainty. For example, transmission models may require data on sexual behavior, although such data may have been previously collected to investigate HIV control strategies [27].

However, simpler models do not guarantee reduced uncertainty, and may indeed increase uncertainty, because certain aspects of the disease or intervention are not explicitly incorporated. Hence, policy-makers short on both data and technical expertise could focus on more basic questions that can be robustly answered with simpler models, and should consider whether their policy decision can also be informed by adapting insights from other settings in which sophisticated modeling has been performed with richer data. For example, countries that have yet to introduce HPV vaccination should first assess the cost-effectiveness of routine vaccination of girls in early adolescence, which often can be addressed using relatively simple models (see Question 4 above). Assessing the cost-effectiveness of male vaccination requires more technically demanding analyses, so countries may need to draw insights from existing studies in high-income [8,28] and middle-income [16] countries, which suggest that initially focusing on increasing vaccine uptake in girls is likely to be a more efficient way to reduce cervical-cancer incidence. In some cases, however, conclusions drawn in other settings may not apply to low-income countries because of differences in sexual behavior (such as partnership concurrency), demographic structure, HPV type distribution, availability of screening and treatment, and co-factors such as HIV infection.

A fourth consideration is the objective, the intended audience, and the ultimate use of results of the modeling exercise. For instance, using a simple model developed by in-country analysts may build internal capacity for policy modeling and cost-effectiveness analysis. If capacity to develop even a simple model *de novo *is lacking, it may be possible for local analysts to be advised by external experts or to adapt a model developed overseas. For example, models of HPV vaccination in Thailand [14] and South Africa [29] were developed by independent in-country analysts adapting an earlier model from the USA [30]. Many modeling groups are open to adapting their existing models to new settings in collaboration with in-country analysts [6]. Another alternative is to develop a regional network of expertise in using a particular model, such as the ProVac initiative in Latin America [4] and the PREHDICT (Prevention Strategies for HPV-related Diseases in European Countries) initiative in Europe [31]. Over time, developing in-country capacity may facilitate more informed policy analyses and greater use of evidence in decision-making. Even a model developed overseas may strengthen capacity if it is parameterized and interpreted by in-country analysts in such a way that they gain knowledge of its design. As these analysts grow more experienced, more complex features such as sexual partnerships, demographic change, and co-factors such as HIV infection can be added. Previous experience from HIV modeling suggests that it may be efficient to build simple models with sufficient flexibility to incorporate added levels of complexity as new questions, evidence, and capabilities emerge [32].

In addition, models constructed locally (or in partnership with foreign expertise) help to engage policy-makers and program managers throughout the analytic process, potentially leading to a deeper understanding of the local drivers of health impact and cost, highlighting the value of data, and increasing awareness of data gaps. Similar engagement might be reached when a complex model is accompanied by an easy-to-use interface that local stakeholders can parameterize themselves. However, results from simple models may be met with less skepticism because their structure, inputs and assumptions are generally more transparent to end users. Lastly, policy-makers and program managers may feel more ownership of analyses conducted locally, so the influence of the results may weigh more heavily in the decision-making process.

## Question 6: How should uncertainty be captured?

Models must convey methodological, structural, and parameter uncertainty [33]. These respectively arise from different choices made about the analytic methods used (for example, payer or societal perspective, discounting rates), underlying model assumptions, and model parameters.

Parameter uncertainty arises when the available input data give only limited information about the 'true' value of the model parameters. This is particularly important in settings where available data are limited. Some health technology authorities such as the National Institute for Health and Clinical Excellence (NICE) in the UK recommend the use of probabilistic sensitivity analysis to study parameter uncertainty. This involves specifying a probability distribution to represent the uncertainty of each model parameter, and then propagating this uncertainty to the outcomes of the analysis (such as the cost-effectiveness ratio). However, even in developed countries, only a few HPV modelers have taken this approach [8]. This may be because HPV models usually contain many parameters whose uncertainty distributions are hard to quantify, and whose values are dependent on each other in ways that require computationally demanding methods to capture. Instead, many cost-effectiveness analyses rely on univariate sensitivity analyses instead, where each parameter is varied within a fixed range and the others kept at their base-case values. Such an approach may still give insight, but does not convey the total amount of uncertainty caused by all parameters.

Structural uncertainty in cervical-cancer models can arise from model representations of the disease's natural history (such as natural immunity mechanisms, progression from neoplasia to cancer, interaction between HPV types, and reactivation of latent infections), effectiveness of interventions (such as the long-term efficacy of vaccination, the effect of vaccine boosting, and the sensitivity of screening tests for detecting glandular lesions) and population behavior (such as screening attendance, vaccine uptake, and sexual behavior). In static models, the effect of herd immunity is uncertain because it is not included in the model. If there are no data to inform such assumptions, structural uncertainty is explored by constructing different scenarios representing alternative assumptions. Ideally, the scenarios should be informed by eliciting expert opinions, and in some cases, this may allow explicit quantification of the uncertainty around particular assumptions.

Methodological uncertainty is ideally addressed by having a 'reference case' specifying the methodological choices that should be made for all health economic evaluations in a country. If this is unavailable, international guidelines (such as WHO recommendations [12]) can be used to ensure comparability with evaluations in other countries, but analysts should also show the consequences of making different methodological choices. Some methodological choices (such as discount rates) are based on national considerations such as income growth rates, so international reference cases may not always be appropriate.

## Question 7: How should model results be communicated?

Whereas simpler models are likely to have more uncertainty, complex models may be more difficult to explain. The most important outcome of a decision analysis is identifying the choice most likely to bring the greatest benefit (the expected utility-maximizing choice) under base-case assumptions [34]. However, assumptions and uncertainties around the outcome must be communicated so that options to improve data or analysis quality can be considered, and the credibility of such analyses is not threatened if the most probable outcomes do not materialize. Unfortunately, when results are presented to decision-makers, the assumptions, caveats, and sensitivity analyses are often greatly simplified. To avoid this, communication should be initiated early as an ongoing process in which key model assumptions are discussed, and their face validity evaluated to inform later model iterations.

Not all outcomes used in economic evaluations (such as cost per DALY prevented) are fully appreciated by decision-makers. Outcomes in clinical units such as morbidity and mortality averted may be most appreciated by clinicians, whereas financial planners may want intervention costs, amounts of averted spending, and the effects on tax receipts and public sector budgets. However, for many such metrics, there is no standard way of comparing between different types of interventions (such as vaccination and spending on acute care). Hence, the opportunity cost of an investment should be made clear; for example, the value of other investments that may be displaced as a result of vaccine introduction.

Other analysts comprise the second audience for communicating model assumptions and results, so that they can provide technical peer review in assessing its validity. Sufficient detail should be provided to allow full reproducibility by capable modelers, and a good understanding of how the model works without having to reproduce it. Although this should in principle be more straightforward for simpler models, a simple model is not necessarily 'transparent' if it is ambiguously documented. A recent discussion paper by the international HPV modeling community suggested that publications of model results should show a variety of measures (such as intermediate disease endpoints, sources of parameters, and methods used to calibrate them) even if they are not directly relevant to the policy decision, to facilitate their appraisal by other modelers [35]. In many cases, this will require the use of online technical appendices due to print journals' word limits.

## Summary

Economic analyses are important tools for facilitating evidence-based decision-making about HPV vaccination. Selecting the appropriate analysis is essential to ensure that all the important features of the decision problem are correctly represented. However, analyses should not be more complex than necessary, as simpler analyses can enable model development by local analysts and greater ownership of the results by local stakeholders. On a research level, a priority for future work is investigating the effect that key behavioral determinants, demographic changes, and disease co-factors important in low-income settings have on model results, so that insights from models of HPV vaccination in HICs can be made more widely applicable.

## Appendix: how the guidelines were formulated

### Panel selection

The guideline development process was initiated by the WHO Initiative for Vaccine Research (IVR) of the Department of Immunization, Vaccines and Biologicals (IVB). In his capacity as senior lead economist for the IVR, RH invited MJ, CL, MB, AL, JB and JK to participate in a technical experts group that convened at the 27th International Papillomavirus Conference on September 17 to 22, 2011. These individuals have an extensive publication record, technical expertise and/or work experience in 1) modeling and economic analysis of HPV vaccination in low/middle-income countries, and 2) contributing to methodological advances in HPV modeling. SR was selected on recommendation of the initial participants, because of his involvement in the CerviVac initiative, a large consortium led by the Pan American Health Organization (PAHO) developing HPV models for LMICs that was not otherwise represented in the panel.

### Reaching recommendations

The WHO initiated discussions during the International Papillomavirus Conference, and asked this group of experts to discuss and outline the issues to be addressed with respect to economic evaluations of HPV vaccination in LMICs, and the key principles to use in addressing them. These were developed further during a teleconference and a face-to-face expert meeting hosted by the WHO in December 2011. An outline of the discussions was drawn up and agreed on by all participants. Participants were then assigned sections of the paper to draft. Each section then received comments from all the other participants. The comments were collated and harmonized by MJ, and sent out for further comments until complete consensus was reached. In total, four iterations of this editing cycle were made.

### Incorporating differing views

The two areas in which participants had differing views were 1) the need for simple versus complex models and 2) the need for a partial analysis (costs or epidemiological outcomes only) versus a full economic analysis. To reconcile these views, a summary of different views was drawn up by MJ and agreed on by all participants. Two participants (SR and RH) were asked to write sections on the complexity and scope of models, respectively. CL and AL were also asked to expand upon when an incremental analysis would be required versus a full economic analysis. These sections then went through the four editing iterations described above until complete consensus was reached. Hence, the final versions incorporated the views of all participants. At the end of the process, there were no further unresolved issues.

### Strengths and limitations of the process

A key strength of this process is bringing together analysts with different skills (in epidemiology, costing, engaging with policy-makers, and methodological development) who are not often involved in working together. By harmonizing different views and opinions, we have been able to produce more nuanced guidelines. One limitation of the process was that participant selection and discussion of topics was not performed using a systematic framework, because the purpose was to produce practical guidelines for field use rather than to propose a comprehensive set of principles to be adopted universally. The way in which consensus was reached (via iterative discussions by a nominated panel of experts) is similar to the methods used by other WHO technical advisory groups such as its Immunization and Vaccines related Implementation Research (IVIR) advisory committee.

## Abbreviations

HPV: Human papillomavirus; HICs: High income countries; LMICs: Low and middle-income countries; WHO: World Health Organization.

## Competing interests

MB has consulted and received reimbursement for travel expenses from Merck Frosst and GlaxoSmithKline. JB's institute received research grants from GlaxoSmithKline in 2007-2010 and incidental consultancy fees from Sanofi Pasteur. None of the other authors has any known competing interests.

## Authors' contributions

RH and MJ conceived the manuscript. All authors contributed towards writing the manuscript and all authors have read and approved the manuscript for publication.

## Acknowledgements

RH is a staff member of the World Health Organization. The views expressed is that of the author and do not necessarily represent the views of the World Health Organization

## Pre-publication history

The pre-publication history for this paper can be accessed here:

http://www.biomedcentral.com/1741-7015/11/23/prepub

## References

[B1] World Health Organization Human papillomavirus vaccines. WHO position paper Wkly Epidemiol Rec 2009 84 118 131 19360985

[B2] Hutubessy R Henao AM Namgyal P Moorthy V Hombach J Results from evaluations of models and cost-effectiveness tools to support introduction decisions for new vaccines need critical appraisal BMC Med 2011 9 55 10.1186/1741-7015-9-55 21569407 PMC3117725

[B3] Yothasamut J Tantivess S Teerawattananon Y Using economic evaluation in policy decision-making in Asian countries: mission impossible or mission probable? Value Health 2009 12 Suppl 3 S26 S30 20586976 10.1111/j.1524-4733.2009.00623.x

[B4] Andrus JK Toscano CM Lewis M Oliveira L Ropero AM Davila M Fitzsimmons JW A model for enhancing evidence-based capacity to make informed policy decisions on the introduction of new vaccines in the Americas: PAHO's ProVac initiative Public Health Rep 2007 122 811 816 18051674 10.1177/003335490712200613 PMC1997250

[B5] Iglesias CP Drummond MF Rovira J Health-care decision-making processes in Latin America: problems and prospects for the use of economic evaluation Int J Technol Assess Health Care 2005 21 1 14 15736509 10.1017/s0266462305050014

[B6] Jit M Demarteau N Elbasha E Ginsberg G Kim J Praditsitthikorn N Sinanovic E Hutubessy R Human papillomavirus vaccine introduction in low-income and middle-income countries: guidance on the use of cost-effectiveness models BMC Med 2011 9 54 10.1186/1741-7015-9-54 21569406 PMC3123559

[B7] Kim JJ Brisson M Edmunds WJ Goldie SJ Modeling cervical cancer prevention in developed countries Vaccine 2008 26 Suppl 10 K76 K86 18847560 10.1016/j.vaccine.2008.06.009 PMC2769256

[B8] Brisson M Van d V Boily MC Economic evaluation of human papillomavirus vaccination in developed countries Public Health Genomics 2009 12 343 351 10.1159/000214924 19684446

[B9] Levin CE Van Minh H Odaga J Sarit Rout S Nguyen Thi Ngoc D Menezes L Mendoza Araujo M La Montagne DS Incremental costs of strategies to deliver human papillomavirus vaccine to young adolescent girls in India, Peru, Uganda and Viet Nam Bull World Health Organ 2012 in press 10.2471/BLT.12.113837 PMC3738308 23940406

[B10] Goldie SJ O'Shea M Campos NG Diaz M Sweet S Kim SY Health and economic outcomes of HPV 16,18 vaccination in 72 GAVI-eligible countries Vaccine 2008 26 4080 4093 10.1016/j.vaccine.2008.04.053 18550229

[B11] Moher D Liberati A Tetzlaff J Altman DG Preferred reporting items for systematic reviews and meta-analyses: the PRISMA statement PLoS Med 2009 6 e1000097 10.1371/journal.pmed.1000097 19621072 PMC2707599

[B12] Walker DG Hutubessy R Beutels P WHO Guide for standardisation of economic evaluations of immunization programmes Vaccine 2010 28 2356 2359 10.1016/j.vaccine.2009.06.035 19567247

[B13] Tracy L Gaff HD Burgess C Sow S Gravitt PE Tracy JK Estimating the impact of human papillomavirus (HPV) vaccination on HPV prevalence and cervical cancer incidence in Mali Clin Infect Dis 2011 52 641 645 10.1093/cid/ciq190 21252142 PMC3106240

[B14] Praditsitthikorn N Teerawattananon Y Tantivess S Limwattananon S Riewpaiboon A' Chichareon S Ieumwananonthachai N Tangcharoensathien V Economic evaluation of policy options for prevention and control of cervical cancer in Thailand Pharmacoeconomics 2011 29 781 806 10.2165/11586560-000000000-00000 21838332

[B15] Ginsberg GM Edejer TT Lauer JA Sepulveda C Screening, prevention and treatment of cervical cancer -- a global and regional generalized cost-effectiveness analysis Vaccine 2009 27 6060 6079 10.1016/j.vaccine.2009.07.026 19647813

[B16] Kim JJ Andres-Beck B Goldie SJ The value of including boys in an HPV vaccination programme: a cost-effectiveness analysis in a low-resource setting Br J Cancer 2007 97 1322 1328 10.1038/sj.bjc.6604023 17923869 PMC2360471

[B17] Senouci K Blau J Nyambat B Coumba FP Gautier L Da Silva A Favorov MO Clemens JD Stoeckel P Gessner BD The Supporting Independent Immunization and Vaccine Advisory Committees (SIVAC) initiative: a country-driven, multi-partner program to support evidence-based decision making Vaccine 2010 28 Suppl 1 A26 A30 20412992 10.1016/j.vaccine.2010.02.028

[B18] Mauskopf JA Sullivan SD Annemans L Caro J Mullins CD Nuijten M Orlewska E Watkins J Trueman P Principles of good practice for budget impact analysis: report of the ISPOR Task Force on good research practices--budget impact analysis Value Health 2007 10 336 347 10.1111/j.1524-4733.2007.00187.x 17888098

[B19] Tan-Torres Edejer T Baltussen R Adam T Hutubessy R Acharya A Evans DB Murray CJLe Making choices in health: WHO guide to cost-effectiveness analysis. Geneva: World Health Organisation; 2003 2003 Geneva, World Health Organisation

[B20] Van d V Brisson M Boily MC Understanding differences in predictions of HPV vaccine effectiveness: A comparative model-based analysis Vaccine 2010 28 5473 5484 10.1016/j.vaccine.2010.05.056 20573580

[B21] Bogaards JA Coupe VM Xiridou M Meijer CJ Wallinga J Berkhof J Long-term impact of human papillomavirus vaccination on infection rates, cervical abnormalities, and cancer incidence Epidemiology 2011 22 505 515 10.1097/EDE.0b013e31821d107b 21540743

[B22] Jit M Chapman R Hughes O Choi YH Comparing bivalent and quadrivalent human papillomavirus vaccines: economic evaluation based on transmission model BMJ 2011 343 d5775 10.1136/bmj.d5775 21951758 PMC3181234

[B23] Dee A Howell F A cost-utility analysis of adding a bivalent or quadrivalent HPV vaccine to the Irish cervical screening programme Eur J Public Health 2010 20 213 219 10.1093/eurpub/ckp141 19864366

[B24] Brisson M Van d V De Wals P Boily MC The potential cost-effectiveness of prophylactic human papillomavirus vaccines in Canada Vaccine 2007 25 5399 5408 10.1016/j.vaccine.2007.04.086 17561316

[B25] Malagon T Drolet M Boily MC Franco EL Jit M Brisson J Brisson M Cross-protective efficacy of two human papillomavirus vaccines: a systematic review and meta-analysis Lancet Infect Dis 2012 10.1016/S1473-3099(12)70187-1 22920953

[B26] Brennan A Chick SE Davies R A taxonomy of model structures for economic evaluation of health technologies Health Econ 2006 15 1295 1310 10.1002/hec.1148 16941543

[B27] Wellings K Nanchahal K Macdowall W McManus S Erens B Mercer CH Johnson AM Copas AJ Korovessis C Fenton KA Sexual behaviour in Britain: early heterosexual experience Lancet 2001 358 1843 1850 10.1016/S0140-6736(01)06885-4 11741623

[B28] Bogaards JA Kretzschmar M Xiridou M Meijer CJ Berkhof J Wallinga J Sex-specific immunization for sexually transmitted infections such as human papillomavirus: insights from mathematical models PLoS Med 2011 8 e1001147 10.1371/journal.pmed.1001147 22205887 PMC3243713

[B29] Sinanovic E Moodley J Barone MA Mall S Cleary S Harries J The potential cost-effectiveness of adding a human papillomavirus vaccine to the cervical cancer screening programme in South Africa Vaccine 2009 27 6196 6202 10.1016/j.vaccine.2009.08.004 19698807

[B30] Kulasingam SL Myers ER Potential health and economic impact of adding a human papillomavirus vaccine to screening programs JAMA 2003 290 781 789 10.1001/jama.290.6.781 12915431

[B31] health-economic Modelling of Prevention STrategies for Hpv-related Diseases in European Countries http://cordis.europa.eu/search/index.cfm?fuseaction=proj.document&PJ_RCN=11314364 Accessed on 29 November 2012

[B32] Lasry A Richter A Lutscher F Recommendations for increasing the use of HIV/AIDS resource allocation models BMC Public Health 2009 9 Suppl 1 S8 10.1186/1471-2458-9-S1-S8 19922692 PMC2779510

[B33] Bilcke J Beutels P Brisson M Jit M Accounting for methodological, structural, and parameter uncertainty in decision-analytic models: a practical guide Med Decis Making 2011 31 675 692 10.1177/0272989X11409240 21653805

[B34] Claxton K The irrelevance of inference: a decision-making approach to the stochastic evaluation of health care technologies J Health Econ 1999 18 341 364 10.1016/S0167-6296(98)00039-3 10537899

[B35] Craig BM Brisson M Chesson H Giuliano AR Jit M Proceedings of the Modeling Evidence in HPV Pre-Conference Workshop in Malmo, Sweden, May 9-10, 2009 Clin Ther 2010 32 1546 1564 10.1016/j.clinthera.2010.06.017 20728767 PMC4095755

